# Innovative atherectomy device for treatment of iliac and popliteal lesions in patients with critical ischemic stage of PAOD

**DOI:** 10.1186/s42155-024-00440-y

**Published:** 2024-03-08

**Authors:** Giel G. Koning, Rüdiger Möller, Ahmed Algharib

**Affiliations:** Department of Vascular and Endovascular Surgery, Euregio Hospital, A Schweitzer Straße 10, 48527 Nordhorn, Niedersachsen, Germany

To the Editor,

With great interest, we have read the articles by our esteemed colleagues J. Teßarek et al. titled “*Atherectomy with a novel device offering wider options*” published in the Zentralblatt für Chirurgie (2023) [1] and their interesting paper in CVIR Endovascular, entitled: “*Safety and effectiveness of ByCross rotational atherectomy and aspiration device: a prospective multicenter pre-market approval study*“ (2023) [2]. In this letter, we wish to share our experiences here in Nordhorn and our opinion with some future perspectives on this device with all CVIR-readers.

Atherectomy is a method for eliminating persistent, recurring constrictions in arteries caused by atherosclerosis, leading to typical symptoms in patients with peripheral arterial occlusive disease (PAOD). A special atherectomy catheter is used to remove the firmly adhering occlusive substances such as plaques. There are various configurations of atherectomy catheters, all involving the physical removal of deposits. The described device aspirates the removed substance, crushes it, and continuously transports it out through the catheter. The advantages of atherectomy with such catheters are the low invasiveness of the procedure, typically performed through a puncture in the Femoral artery and the introduction of the catheter via a sheath in the Seldinger technique, as well as the rapid restoration of blood flow in narrowed or even occluded vascular segments. This is especially true when atherothrombectomy catheters are used, removing blood clots and vascular wall deposits in a single procedure. The complete or partial removal of vascular deposits can also benefit adjunctive procedures such as balloon dilatation (PTA) and/or stenting, as the removal of material reduces the need to stretch the vascular wall, potentially leading to fewer complications and recurrences. Catheter-guided atherectomy has been under discussion for several years, though conclusions were controversial. However, recent study data demonstrated the feasibility of multifunctional atherectomy devices for complex lesion morphologies with moderate material consumption. This presents an alternative to bypass surgery, although a direct comparison is lacking.

The results of the device’s approval study were compared concerning the scope of application, manufacturer-defined limitations, success and complication rates, and instructions for use with technical and clinical data from various atherectomy systems [2]. The results of the 6-month follow-up showed a very satisfactory revascularization rate. The atherectomy device is in a certain way an atherectomy, thrombectomy, and transit device without investment costs and has a broader scope of application as an alternative to bypass surgery [2].

Meanwhile, we have gained experience with this device and use it, in addition, for both Iliac and Popliteal artery occlusions, achieving satisfactory results from both the patient’s as well as the surgeon‘s perspective. We propose a new multi-center study focusing on the Iliac area as well as the Popliteal area to confirm or reject these initial promising results and also to verify and report these so-called “Patient Reported Outcomes” using the method of a focused group discussion [3]. Currently, this atherectomy expertise with the mentioned device is at the fifth level of evidence-based medicine [4]. Therefore, these first reported results should be interpreted cautiously and, in our opinion, should currently be used by experienced endovascular hands. Until experience is achieved the ESVS-guidelines should not be abandoned. We look forward to further objective study results of this device with great interest!

## Data Availability

N/A.

## Abbreviations

PAOD: peripheral arterial occlusive disease
ESVS: European Society for Vascular Surgery
CVIR: cardiovascular and interventional Radiology
PTA: percutaneous transluminal angioplasty
